# The fly liquid-food electroshock assay (FLEA) suggests opposite roles for neuropeptide F in avoidance of bitterness and shock

**DOI:** 10.1186/s12915-021-00969-7

**Published:** 2021-02-16

**Authors:** Puskar Mishra, Shany E. Yang, Austin B. Montgomery, Addison R. Reed, Aylin R. Rodan, Adrian Rothenfluh

**Affiliations:** 1 Molecular Medicine Program, University of Utah, Salt Lake City, UT USA; 2 Department of Bioengineering, University of Utah, Salt Lake City, UT USA; 3 Department of Internal Medicine, Division of Nephrology & Hypertension, University of Utah, Salt Lake City, UT USA; 4 Department of Human Genetics, University of Utah, Salt Lake City, UT USA; 5 Medical Service, Veterans Affairs Salt Lake City Health Care System, University of Utah, Salt Lake City, UT USA; 6 Department of Psychiatry, University of Utah, Salt Lake City, UT USA; 7 Department of Neurobiology, University of Utah, Salt Lake City, UT USA

## Abstract

**Background:**

Proper regulation of feeding is important for an organism’s well-being and survival and involves a motivational component directing the search for food. Dissecting the molecular and neural mechanisms of motivated feeding behavior requires assays that allow quantification of both motivation and food intake. Measurements of motivated behavior usually involve assessing physical effort or overcoming an aversive stimulus. Food intake in *Drosophila* can be determined in a number of ways, including by measuring the time a fly’s proboscis interacts with a food source associated with an electrical current in the fly liquid-food interaction counter (FLIC). Here, we show that electrical current flowing through flies during this interaction is aversive, and we describe a modified assay to measure motivation in *Drosophila*.

**Results:**

Food intake is reduced during the interaction with FLIC when the electrical current is turned on, which provides a confounding variable in studies of motivated behavior. Based on the FLIC, we engineer a novel assay, the fly liquid-food electroshock assay (FLEA), which allows for current adjustments for each feeding well. Using the FLEA, we show that both external incentives and internal motivational state can serve as drivers for flies to overcome higher current (electric shock) to obtain superior food. Unlike similar assays in which bitterness is the aversive stimulus for the fly to overcome, we show that current perception is not discounted as flies become more food-deprived. Finally, we use genetically manipulated flies to show that neuropeptide F, an orthologue of mammalian NPY previously implicated in regulation of feeding motivation, is required for sensory processing of electrical current.

**Conclusion:**

The FLEA is therefore a novel assay to accurately measure incentive motivation in *Drosophila*. Using the FLEA, we also show that neuropeptide F is required for proper perception or processing of an electroshock, a novel function for this neuropeptide involved in the processing of external and internal stimuli.

**Supplementary Information:**

The online version contains supplementary material available at 10.1186/s12915-021-00969-7.

## Background

Motivation can be regarded as an organism’s goal-directed quest for change, for example the search for food in the face of starvation. Numerous human conditions show aberrations in motivated behaviors, such as psychiatric disorders like depression or addiction, but also neurodegenerative diseases such as Parkinson’s or dementia [1]. The mechanistic dissection of the neural and molecular mechanisms of motivated behaviors is thus of considerable relevance to human health. Assays to measure motivation in animal models generally involve them exerting physical effort, or overcoming an aversive stimulus, such as walking across an electrified grid. When the external incentive is increased, rats, for example, will cross a grid that delivers a larger electric shock [2]. In addition to external incentives acting as motivators, the other main component to motivational behavior is the internal state and resulting drive of the animal [2]. The integration and valuation of internal drive and external incentive is what stirs the animal into goal-directed action. Feeding is one of the fundamental actions in animals and is normally under tight regulation to keep an organism’s energy expenditure and stores in balance [3]. Eating disorders are common human dysregulations of feeding and are still not well understood at the molecular and neural level [4]. Since all animals regulate their food intake, model organisms can help in the dissection of the mechanisms regulating the motivation of feeding behavior.

The vinegar fly, *Drosophila melanogaster*, has been a genetic model organism for over 100 years, and numerous assays exist to determine a fly’s feeding behavior. These include measuring food consumption from a tiny capillary [5] or lacing the food with quantifiable substances, such as dyes [6, 7], radioactive compounds [8], or even oligonucleotides [9]. Recently, additional assays have been developed that rely on feeding flies closing an electrical circuit that allows the interaction between fly and food to be measured in intensity and duration [10]. Even though the latter assays do not measure actual ingestion, the time spent interacting with the food correlates well with the amount ingested [11]. Thus, these assays are valuable additions due to their wide temporal range—from milliseconds to days—over which they can record feeding events. Some of the above feeding assays have been coupled with bitter substances, in order to determine flies’ willingness to overcome aversion to get to food, resulting in a measure of their feeding motivation [12]. However, as flies become starved, their peripheral perception of bitterness decreases [13, 14]. Thus, seemingly increased motivation can be caused, at least in part, by the decreased perception of the aversive stimulus in the first place, thereby confounding the quantitative assessment of flies’ motivation.

Here, we develop a novel feeding assay based on the fly liquid-food interaction counter (FLIC [10];). Our novel assay allows for individual feeding wells to be paired with different amounts of current delivered, enabling us to ask what variables motivate flies to overcome a higher current to obtain food. We show that external incentives and internal drive both act as feeding motivators. Lastly, we find that neuropeptide F (NPF) also plays a role, albeit in the perception of the electrical current itself, thus revealing a novel function for this neuropeptide, previously linked to feeding motivation [15, 16].

## Results

The fly liquid-food interaction counter (FLIC) is a *Drosophila* feeding assay that allows for continuous online feeding monitoring [10]. When flies standing on a metal plate make contact with the liquid food, they complete an electrical circuit, which allows for precise measurement of the duration the flies interact with the food. In addition, the amplitude of the signal depends on whether flies touch the food with their legs (lower amplitude “leg events”) or engage in food consumption using their proboscis (higher amplitude “proboscis events”).

**Fig. 1:**
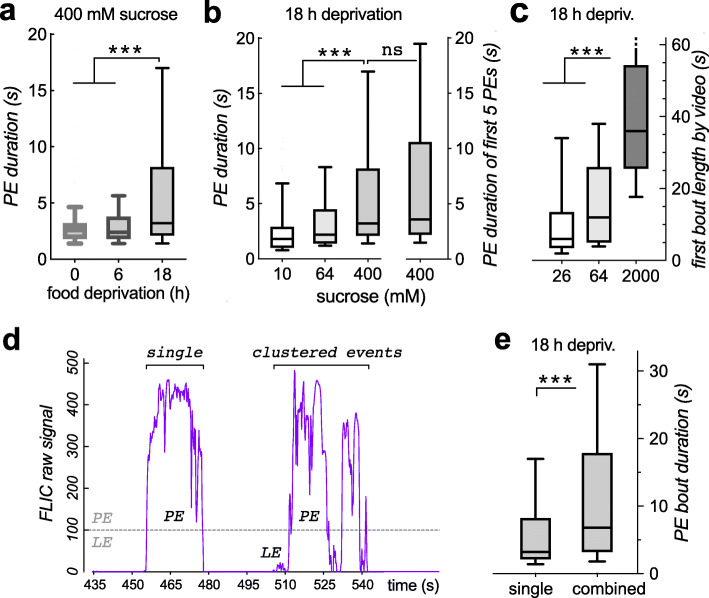
Feeding-event duration measured in the FLIC. **a** Longer food deprivation times result in significantly longer proboscis events (PEs) on 400 mM sucrose (***p < 0.001, Mann-Whitney test with Dunn’s correction, *n* = 233, 233, 349 events. Here, and in the following panels, the first 15 min of FLIC events were analyzed, unless specified otherwise. Ten male flies were used per arena, with 6–8 arenas analyzed for each FLIC experiment. The FLIC uses a 10-MΩ current-limiting resistor). **b** PE durations are longer when flies are offered higher sucrose concentrations (left side; ***p < 0.0001 *n* = 75, 137, 349 events from 6 to 8 arenas). If only the first 5 PEs per feeding well are analyzed (right side), the median PE duration increases slightly, but not significantly (ns not significant, *p* = 0.37, *n* = 349, 143 events). **c** Length of first feeding bout as measured by video recording. The median length of bouts, here defined as uninterrupted engagement with the liquid food, increase with sucrose concentration offered (***p < 0.0001, *n* = 45, 58, 46; one fly per *n*). Note that the bouts are considerably longer when compared to measurements in the FLIC (**a**, **b**). **d** Example data from a FLIC well (400 mM sucrose, 18 h deprivation). Some PEs occur in single isolation (left). Others come in clustered bouts of PEs (right), together with leg events (LE, signal amplitude < 100 over baseline), that follow in close succession. **e** When PEs that occur in groups of events (with an inter-event interval of less than 5 s) are grouped into combined feeding events, the median bout length increases significantly compared to analyzing all PEs as single events (as done in **a**; *p* < 0.0001, *n* = 349, 188 events on 400 mM sucrose, 8 arenas; all data are shown as medians with quartile boxes and 10–90 percentile whiskers). The data for this and all subsequent figures can be accessed in Additional File 4: FLEA_Data_File.xlsx

We were interested in measuring feeding-event duration in the FLIC. To that extent, we changed two variables that should have predictable effects on feeding events: sucrose concentration offered and prior food deprivation. When we determined the median duration of proboscis events in a 15-min FLIC assay, we found that the duration increased with the length of prior food deprivation (Fig. 1a). Similarly, proboscis event duration also increased when we increased the amount of sucrose offered (Fig. 1b, left). After an 18-h food deprivation, the median proboscis event length was 3.2 s on 400 mM sucrose. We similarly food-deprived flies for 18 h and then filmed them feeding on liquid sucrose in a petri dish. When we determined the duration of the first feeding bout, we again saw that bout duration increased with the amount of sucrose offered (Fig. 1c). We also found that the median first bout lasted 12 s on 64 mM sucrose, considerably longer than in the FLIC on the higher, 400 mM, sucrose concentration.

To try to understand this discrepancy in event/bout duration, we first tested the hypothesis that later events in the 15-min FLIC assay were shorter, as flies became satiated, thus lowering the measured median event length. Analyzing only the first 5 events in the FLIC—from a total of 10 flies—revealed a small, but non-significant increase in event duration (Fig. 1b, right). We thus rejected shortness of later events as the cause for the event duration difference in the FLIC versus feeding in a dish. In our free-feeding filming experiment, we counted a brief disengagement of the proboscis, followed by immediate re-engagement of the proboscis with the food, as being part of one and the same feeding bout, reasoning that there was no interruption of the feeding by a distinctly different behavior. In the FLIC, proboscis interaction-events sometimes appear as long and isolated, and sometimes in clusters, interspersed with leg interactions (Fig. 1d). When we examined the frequency distribution of the event duration, we saw an obvious inflection point at 5 s (Additional file 1: Fig. S1). We therefore grouped interaction-events containing at least one proboscis interaction which were closer than 5 s into one long bout. Forty-six percent of proboscis events fell within such longer, grouped bouts, and this analysis led to a significant increase in the median bout duration in the FLIC on 400 mM sucrose, from 3.2 to 6.8 s (Fig. 1e). Considering bout structure and grouping is therefore part of the reason why the bout duration in the FLIC is shorter than when free feeding.

**Fig. 2:**
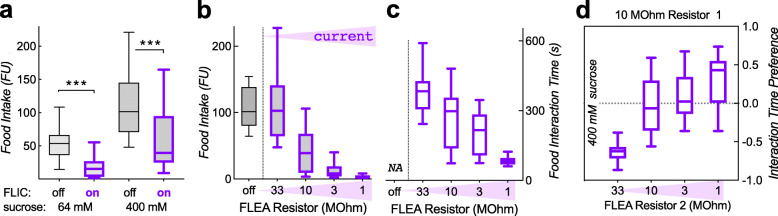
FLIC current is aversive to flies. **a** Turning on FLIC current (10-MΩ resistor) significantly reduces food intake of 18-h food-deprived flies, as measured by fluorescent dye ingestion (FU, fluorescence units measured in plate reader; ***p < 0.0001, *n* = 33, 34, 51, 49 from 12 FLIC arenas; an *n* of 1 is the combined fluorescence from 2 flies). **b** Redesigned FLIC with adjustable current (FLEA) shows a decrease in food intake as a function of current (*p* < 0.0001; one-way Kruskal-Wallis ANOVA, *n* = 39, 32, 36, 28, 29 from 11 to 12 arenas, 2 flies per *n*). **c** Food interaction time, as measured by the FLEA current, is also significantly reduced as the current increases (*p* < 0.0001; *n* = 11, 12, 12, 12 arenas with 10 males each; NA, not available; 18 h deprivation, 400 mM sucrose in **b** and **c**). **d** Flies change their interaction time preference as the current increases in one of two equal-sucrose wells (*p* < 0.0001; *n* = 10, 11, 11, 10 arenas with 10 males each)

However, even the bout-grouped median duration of 6.8 s on 400 mM sucrose in the FLIC (Fig. 1e) was still considerably shorter than the free-feeding median of 12 s on the lower 64-mM sucrose concentration (Fig. 1c). While these data did not compare the exact same measure (first 5 feeding bouts total from 10 flies in the FLIC vs. first feeding bout in filmed single flies), the discrepancy was large enough to consider a third hypothesis, which posited that the current in the FLIC is causing a reduction in proboscis interaction. To test this, we added a fluorescent dye to the sucrose solution in the FLIC and then measured the amount ingested in a plate reader, as we have done before [6]. Indeed, turning on the FLIC current caused a significant reduction in food intake of both 64 and 400 mM sucrose (Fig. 2a). This indicated that the FLIC current is aversive to flies when they are feeding.

The FLIC has a fixed design, which includes a 10-MΩ resistor to limit the current flow when the circuit is closed [10]. We redesigned the FLIC in a way that allowed us to modularly exchange this current-limiting resistor for each food well. Because this new assay also allowed us to increase the current, we named it the FLEA, for fly liquid-food electroshock assay (see Additional file 2: Fig. S2 for setup and circuit board design). We first tested whether altering current flow when flies closed the circuit would have an impact on sucrose intake labeled with a fluorescent dye. As hypothesized, the smaller the resistor, and the higher the current, the lower the amount of food ingested (Fig. 2b). Similarly, the time spent interacting with the food as measured by the current signal in the FLEA was also shorter, the higher the current (Fig. 2c). Proboscis event duration also decreased with higher current, as did the frequency of proboscis (vs. leg only) events (Additional file 3: Fig. S3).

**Fig. 3:**
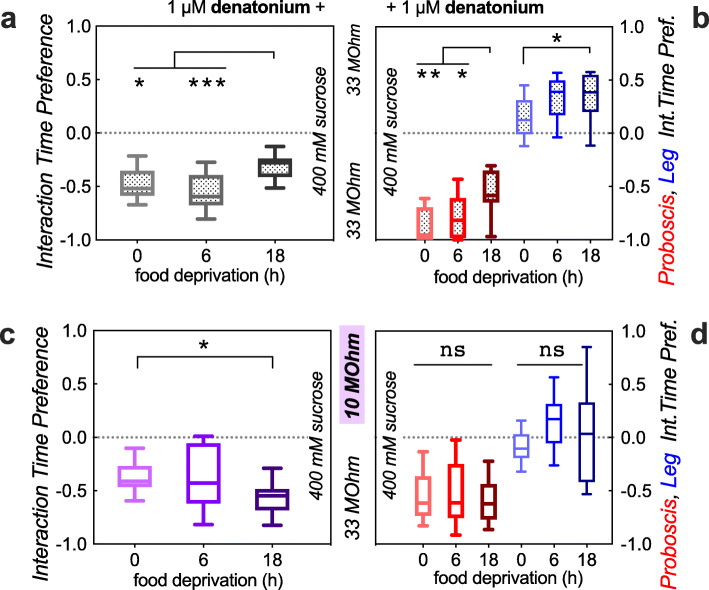
Unlike bitterness, current is not discounted upon food deprivation. **a** 18-h deprived flies show reduced avoidance of denatonium (***p = 0.0011, *p = 0.0495, Kruskal-Wallis test with Dunn’s correction, *n* = 14, 13, 14). **b** Both leg (blue, *p = 0.031) and proboscis (red, **p = 0.0013, *p = 0.039, *n* = 14, 13, 15, 15, 14, 16 arenas of 10 males, here and in all subsequent FLEA panels) interactions show decreased sensitivity to denatonium with increased food deprivation. **c** Current avoidance increases with food deprivation when determining total (proboscis+leg) interaction time (*p = 0.022; *n* = 14, 20, 20 arenas). **d** However, neither proboscis (red, ns not significant, *p* = 0.73) nor leg (blue, ns *p* = 0.06, *n* = 14, 20, 21, 15, 20, 21 arenas) interaction time preference changes with food deprivation. The apparent decrease in total interaction preference in **c** is caused by a shift from leg to proboscis interactions upon food deprivation, lowering the combined Preference Index

We reasoned that we might make use of the current as an aversive stimulus to overcome and designed the FLEA as a 2-choice assay where one choice goes with higher current. We first tested whether flies would prefer to interact with food that was paired with the lesser current, while food quality remained equal. Indeed, the flies’ interaction preference changed as we altered the current-limiting resistor in one of the two wells (Fig. 2d), suggesting that we might be able to use the FLEA as an assay to measure feeding motivation. Such feeding experiments—asking whether flies are willing to overcome an aversive stimulus—have been described using bitter substances mixed in with one of the two feeding solutions [12]. The willingness to overcome bitterness can then serve as a proxy for flies’ feeding motivation. However, an assay including bitterness has a significant confounder: the perception of bitterness depends on the flies’ food deprivation status, with hungry flies showing decreased responses to bitterness [13, 14]. We replicated this by testing flies’ willingness to overcome 1 μM denatonium and indeed found significantly reduced aversion to this bitter substance with longer periods of food deprivation. Flies demonstrated less avoidance (less negative interaction time preference) after 18 h compared with shorter deprivation (Fig. 3a). We also determined the preference for proboscis (feeding) and leg (tasting) interactions separately, and both showed an effect with increased food deprivation (Fig. 3b). This made sense, since there are bitter sensory neurons located on both the legs and the proboscis [14, 17]. Our data thus confirmed that bitterness is discounted by food deprivation. To use the FLEA as an assay for feeding motivation, the perception of the current should not be altered by the duration of prior food deprivation. We therefore performed the same experiment as with denatonium, this time with current as the deterrent, varying the duration of prior food deprivation. Avoidance of the well with higher current increased with deprivation time (Fig. 3c). This would suggest that flies actually become more sensitive to current as they are food deprived for longer. However, when we analyzed the proboscis and leg interaction preference separately, neither of them depended on the duration of food deprivation (Fig. 3d). Flies strongly avoided proboscis interaction with the higher current, while leg interactions were insensitive. This suggested two things: first, in a setting of 10 vs. 33 MΩ (Fig. 3d), the leg-mediated current cannot be perceived, probably because it is considerably smaller than proboscis-mediated current (see Fig. 1d). Second, because food deprivation increases the frequency of proboscis over leg events (as flies are hungry and want to feed), proboscis interactions become more prevalent after 18 h of food deprivation (data not shown, and [13]). As the proboscis interactions are more sensitive to current than the leg interactions, this skews the total (proboscis+leg) interaction preference towards the negative, i.e., lower current well, explaining the apparent increase in sensitivity to current of total event preference with increasing food deprivation (Fig. 3c).

**Fig. 4:**
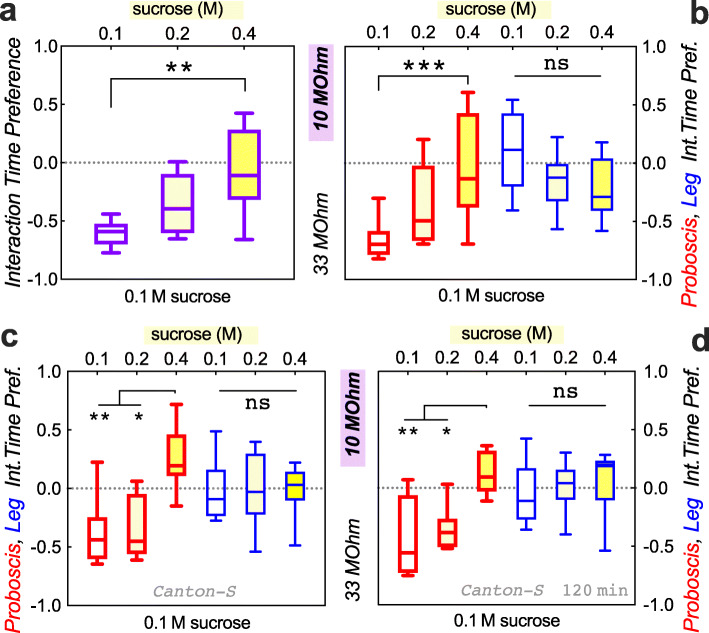
Increased sucrose concentration causes reduced current avoidance in food-deprived flies. **a**–**c** Increasing the sucrose concentration in the food well with higher current reduces flies’ avoidance of that food well. Both total interaction preference (**a**, **p = 0.002, Kruskal-Wallis test with Dunn’s correction; *n* = 10, 12, 12 arenas) and proboscis interaction preference (**b**, ***p = 0.0009, *n* = 11, 12, 12, 12, 11, 11 arenas, w* Berlin flies) increase significantly with sucrose concentration. Proboscis interaction preference also increases in *Canton-S* flies (**c**, **p = 0.0094, *p = 0.0164, *n* = 8, 8, 7, 8, 8, 7 arenas; 15-min analysis here, and in above panels). A 2-h analysis shows the same qualitative result (**d**, **p = 0.0077, *p = 0.0391, *n* = 8, 8, 7, 8, 8, 7 arenas). Flies were food-deprived for 18 h before testing

Because our data suggested that current perception by the proboscis and by the legs is not discounted by food deprivation, we wanted to establish the FLEA as an assay for motivation. We next tested whether an external incentive would induce flies to overcome a higher current. As hypothesized, 18-h food-deprived flies showed less aversion to a higher current when the high-current well contained more sucrose (Fig. 4a, b). To test whether this was a general phenomenon, we assayed a second wild-type strain to our w* Berlin one. Reduced aversion to current when paired with higher sucrose was also observed in *Canton-S* flies, both in our standard 15-min (Fig. 4c) and also in a longer 2-h FLEA assay (Fig. 4c). Thus, flies are willing to overcome current, if enough of an external incentive is paired with it. Furthermore, the FLEA allowed us to assign a value on the incentive, which is the incentive size attractive enough to equal the aversion to a given current, resulting in a preference index of 0. In this experiment, it took a fourfold increase in sucrose concentration to offset the aversion to 10-MΩ current (Fig. 4).

**Fig. 5:**
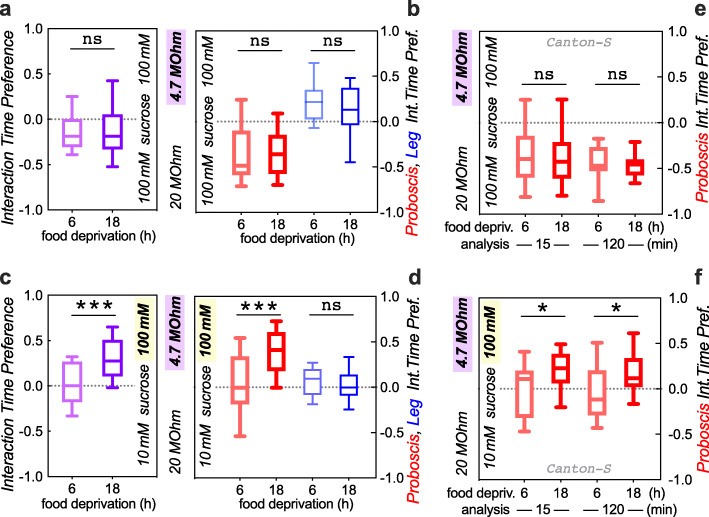
Increased internal feeding drive causes reduced current avoidance. **a** Increasing the duration of food deprivation from 6 to 18 h has no effect on flies’ avoidance of 4.7- vs. 20-MΩ current at equal sucrose (ns *p* = 0.88, Mann-Whitney *U* test; *n* = 29, 30). **b** This was also true for proboscis and leg interaction preference (ns *p* = 0.50 and 0.42, *n* = 27, 28, 27, 28 arenas, 10 w* Berlin flies). **c** The combination of 100 mM sucrose with 4.7-MΩ current became attractive only after 18 h of food deprivation (***p = 0.0005; *n* = 27, 28 arenas). **d** This was also evident in the proboscis interaction preference (***p = 0.0005, *n* = 26, 28), while leg interaction preference remained unchanged (ns *p* = 0.38, *n* = 26, 30). **e**, **f** The same qualitative results are observed with a 15- or 120-min analysis of *Canton-S* flies: no effect of deprivation duration on proboscis interaction preference when sucrose concentrations are equal (**e**, *p* ≥ 0.63, *n* = 10 arenas each). Conversely, proboscis interaction preference increases when higher, 100 mM sucrose was paired with higher, 4.7-MΩ current and *Canton-S* flies were food deprived for 18 h (**f**, 15 min *p = 0.0476, 120 min *p = 0.0283, *n* = 19, 20, 19, 20 arenas)

Next, we wanted to test whether internal drive would induce flies to overcome higher current. To do so, we compared flies that were food-deprived for 6 vs. 18 h, a time difference that has been shown to lead to significant feeding changes [18]. When we first performed this experiment pairing 10 mM sucrose with 33-MΩ current versus 100 mM sucrose with the 10-MΩ current, we found a trend, but no significant effect of food deprivation (data not shown). Using the 33-MΩ resistor leads to a current that does not deter flies from feeding (Fig. 2b, c), thereby setting up a steep gradient against 10-MΩ current. We decided to instead use a resistor pair where both currents were perceptible to the flies. In a choice of these currents, each paired with 100 mM sucrose, flies preferred to interact with food paired with the lower 20-MΩ over the higher 4.7-MΩ current (Fig. 5a). The median preference index in this setting was − 0.19 (Fig. 5a), which was considerably less aversive compared to our prior 33 MΩ vs. 10 MΩ comparisons with equal sucrose, where the preference indices ranged from − 0.41 to − 0.62 (Figs. 2d, 3c, and 4a). This suggested that our 20 vs. 4.7 MΩ setup presented a lesser current gradient than the initial 33 vs. 10 MΩ choice. As before (Fig. 3c, d), the perception of the current in and of itself did not depend on the 6- vs. 18-h duration of food deprivation (Fig. 5a, b). When we next paired 100 mM sucrose with the higher 4.7-MΩ current, this solution was equally palatable to 6-h deprived flies as a 20-MΩ/10-mM pairing. However, after an additional 12 h of food deprivation, the flies preferred the higher current/higher sucrose 4.7-MΩ/100-mM well (Fig. 5c, d), suggesting that increased internal feeding drive caused the flies to be willing to overcome a higher current to obtain better food. This again generalized to a second wild-type strain *Canton-S*, in our standard 15-min and longer 2-h analysis (Fig. 5e, f).

**Fig. 6:**
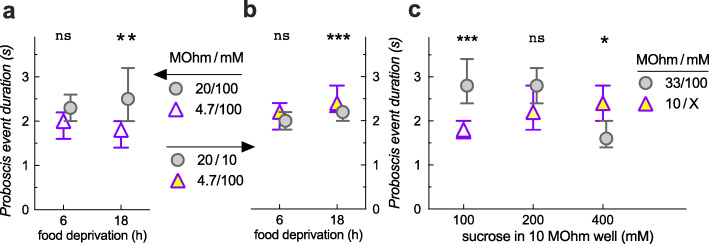
Proboscis event duration changes with various parameters in a choice assay. **a** Event analysis from Fig. 5e, 15 min, reveals that proboscis event duration increases with food deprivation on the low-current well only (For this figure, medians with 95% confidence intervals are shown. For the analysis, the data were log-transformed to run 2-way ANOVAs, see the “Methods” section. Main effect of food well, *p* = 0.0006, but not of deprivation time, *p* = 0.41; event duration is no different between the high- vs. low-current well after 6-h food deprivation, *p* = 0.23, but longer on the low-current 20-MΩ well after 18 h, **p = 0.0016, Sidak’s multiple comparison test, *n* = 136, 75, 84, 116 events from 10 arenas per condition with 10 *Canton-S* males). **b** Analysis of Fig. 5f, 15 min, shows a main effect of well (*p* = 0.041), deprivation (*p* = 0.0012), and interaction (*p* = 0.029, *n* = 239, 209, 331, 368 events from 19 to 20 arenas). There is a slight, but highly significant increase in proboscis event duration on the higher current/higher sucrose well (***p < 0.0001) compared to the lower current/lower sucrose after 18 h of food deprivation. **c** Event analysis from Fig. 4c reveals a main effect of the well (*p* = 0.019), sucrose concentration (*p* = 0.028), and interaction (*p* < 0.001). With equal sucrose, flies had longer event durations on the lower current well (***p < 0.001), but they had longer proboscis event durations on the high-current well when paired with higher, 400 mM sucrose (*p = 0.015). Event duration increased significantly with sugar concentration on the high-current/variable-sucrose well (*p* = 0.006, linear regression), and it decreased significantly on the low-current/constant-sucrose well (*p* < 0.001; *n* = 164, 247, 139, 223, 165, 155 events from 7 to 8 arenas per condition, 10 *Canton-S* males)

We found that proboscis event duration depended on food deprivation (Fig. 1a), sucrose concentration offered (Fig. 1b), and aversive current (Additional file 3: Fig. S3). We therefore wanted to determine how these variables affected proboscis event duration in a 2-choice setting. With equal sucrose, additional food deprivation led to an increase in proboscis event duration on the low-current, but not high-current well (Fig. 6a). In contrast, there was no change in proboscis event duration upon longer deprivation on a low-current/low-sucrose well, while there was a slight, but significant increase on the high-current/high-sucrose well (Fig. 6b). Thus, proboscis event duration changes are dependent on the choice context, and not simply on one of our manipulated variables. This was even more evident when we analyzed proboscis event duration from Fig. 4c, where increasing sucrose was paired with a high-current well. As expected, proboscis event duration increased with higher sucrose concentrations (Fig. 6c). In addition, though, proboscis event duration on the choice-2 low-current/fixed-sucrose well decreased with increasing sucrose in the high-current/variable-sucrose choice-1 well (Fig. 6c). Thus, both the proboscis event duration and the preference indices are reflecting a comparative evaluation of both feeding wells offered—what one would hope for in a 2-choice assay.

**Fig. 7:**
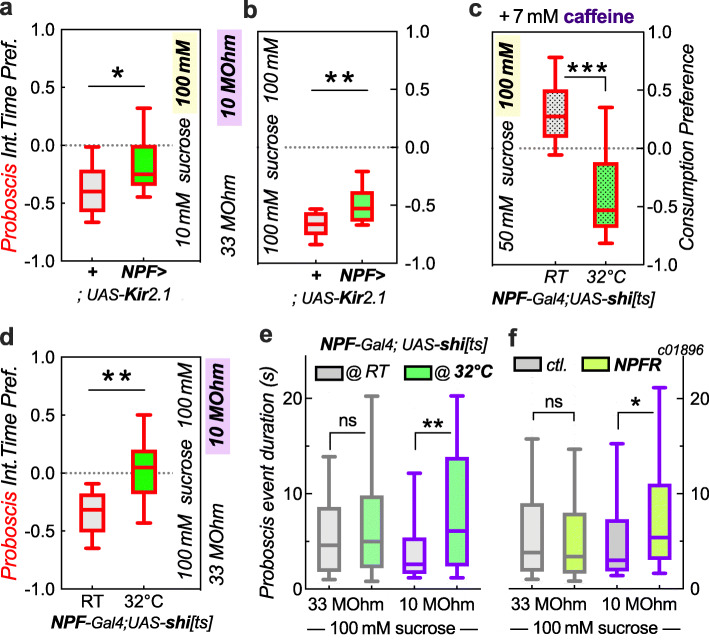
Reduced NPF signaling leads to reduced shock avoidance. **a** Flies with reduced NPF signaling show decreased avoidance of a high-current/high-sucrose combination (*p = 0.017, Mann-Whitney *U* test, *n* = 18 arenas each). **b** These flies also show reduced avoidance of higher current at equal sucrose concentration combination (**p = 0.006, *n* = 14, 13 arenas). **c** 18-h food-deprived flies with reduced NPF signaling show increased avoidance of a bitter caffeine/high-sucrose combination at the restrictive temperature in a fluorescence ingestion choice assay combination (***p < 0.0001, *n* = 16, 15 from 4 feeding choice plates, 30 flies per plate, where an *n* of 1 is the combined fluorescence of 3 flies). **d** These same flies show decreased avoidance of a higher current combination (**p < 0.003, *n* = 9, 11 arenas). **e** Reduced NPF signaling also leads to an increase in median proboscis event length at the restrictive temperature with 10-MΩ current (**p = 0.0012, *n* = 47, 86 events from 8 arenas), but not on the 33-MΩ, imperceptible current well (ns *p* = 0.14, *n* = 103, 77 events from 8 arenas). **f** Similarly, mutation in the NPF receptor leads to increased event duration on the 10-MΩ (**p = 0.007, *n* = 96, 80 events from 8 arenas), but not 33-MΩ well (ns *p* = 0.20, *n* = 255, 271 events from 8 arenas)

Lastly, we wanted to test the role of the neuropeptide F (NPF) in feeding motivation, using our novel FLEA assay. Fly larvae showed an enhanced willingness to ingest bitter food with increased NPF signaling, while reduced NPF signaling made larvae ingest less bitter-laced food [15]. This suggested that NPF is involved in feeding motivation and that NPF signaling might similarly cause flies to overcome higher current to get to better food. To our surprise, when we silenced NPF neurons by overexpression of the inwardly rectifying Kir2.1 channel in *NPF-Gal4* neurons, those flies were more attracted to the higher current side (10 MΩ/100 mM sucrose vs. 33 MΩ/10 mM; Fig. 7a), the opposite result of what we expected. We then tested whether these flies were as sensitive to the current itself as their controls, and we found that they were less deterred by current when presented with equal sucrose in both wells (100 mM, 10 vs. 33 MΩ; Fig. 7b). This suggested that NPF is required for proper perception of electroshock, a function for NPF not previously proposed. We therefore wanted to replicate this finding using *NPF-Gal4* driving a temperature sensitive *shibire*^*ts*^ gene causing neuronal silencing. Larvae carrying *NPF>shi*^*ts*^ were previously shown to be more sensitive to quinine in the food at the restrictive temperature. We first wanted to replicate this finding in adult flies. Using our two-choice fluorescence consumption assay [6], we found that at the control temperature, 18-h food-deprived *NPF>shi*^*ts*^ flies preferred 7 mM caffeine/100 mM sucrose vs. 50 mM sucrose alone. However, at the restrictive 32° temperature, these flies avoided the caffeine/sucrose solution (Fig. 7c), consistent with the proposed model that NPF is required to overcome bitterness in food [15]. We then tested these flies in the FLEA and again found that reduced NPF signaling at the restrictive temperature lowered flies’ avoidance to higher current at equal sucrose (Fig. 6d). This again supported the hypothesis that NPF signaling is involved in the perception of electroshock. Next, we assessed proboscis event duration with varying current. There was no effect of silencing NPF neurons (*NPF>shi*^*ts*^ flies) when the current was imperceptible (33-MΩ resistor). However, at higher currents (10-MΩ resistor), *NPF>shi*^*ts*^ flies showed a significantly increased proboscis event duration at the restrictive temperature (Fig. 7e). This suggested that NPF signaling is required to inform flies of the aversive shock, which induces termination of a feeding event. We also replicated this finding using NPF receptor mutants, *NPFR*^*c01896*^, which also showed significantly longer proboscis event duration when exposed to greater current (10-MΩ resistor), but not on low/imperceptible current (33 MΩ; Fig. 7f). Therefore, three distinct genetic *NPF* manipulations supported the interpretation that NPF signaling is required for proper perception of electroshock.

## Discussion

Here, we describe the FLEA as a novel feeding assay based on the design for the FLIC, where flies touch a liquid food source and complete an electrical circuit, leading to a small current [10]. This allows for the precise measurement of feeding-time interactions and can be used longitudinally, over the course of days [10]. We were interested in more short-term measurements to determine the variables affecting individual feeding events. As expected, feeding events were lengthened with increasing food quality, and prior food deprivation (Fig. 1a, b). Because the absolute durations of these feeding events were considerably smaller than what we observed by filming freely feeding flies (Fig. 1c), we suspected that the FLIC current might actually be aversive to the flies. Indeed, the FLIC current limited by a 10-MΩ resistor caused about a threefold reduction in actual food ingestion (Fig. 2a). Our data also showed that a 33-MΩ current was undetectable by the flies (Fig. 2b), and they preferred to interact with a 33-MΩ feeding well over a 10-MΩ well, at over a 3:1 ratio (Figs. 2d, 3c, and 4a). The negative value of a 10-MΩ current is only offset by a fourfold increase in sucrose concentration (Fig. 4). The current generated from a leg interaction is about 5 times smaller than that for a proboscis interaction (Fig. 1d), and our data suggest that flies cannot detect the 10-MΩ current with their legs (Figs. 3d and 4b). This current difference is of similar magnitude as the 10-MΩ aversive/33-MΩ imperceptible current ratio, and overall our data suggest that while the 10-MΩ FLIC current is close to innocuous, it is aversive to flies touching the food with their proboscis and will report skewed durations of feeding interactions. Higher currents lead to even shorter food interactions and smaller volumes ingested (Fig. 2b, c), and at the highest, and very aversive 1-MΩ current, very few proboscis events were found (Additional file 3: Fig. S3), suggesting that this high current was perceptible to flies touching it with just their legs. But even at that highest current, we never found any evidence of flies being electrocuted. How the flies perceive this electric shock is not known. A genome-wide association study found a number of otherwise unrelated genes to be involved and suggested that those genes might be involved in bristle function [19]. The *pickpocket* gene, which is required for mechanical nociception, was not needed for normal electroshock-induced avoidance memory [20]. It thus remains to be seen what sensory processes are involved in the perception of the FLIC/FLEA-mediated electric current.

Based on these findings, we re-engineered a FLIC-like assay that allows for adjustable currents to be paired with each one of two feeding wells in order to measure flies’ feeding motivation. Prior assays paired one well with a bitter substance, to gauge flies’ motivation to overcome an aversive stimulus while feeding [12]. One confound in this setup is that flies’ devalue bitterness with increasing food deprivation (Fig. 3a, b [13];). This makes sense in the wild, where a (hungry) “beggar can’t be a chooser,” but it also means that assays of feeding motivation relying on bitterness as a deterrent are confounded by the flies’ internal state of satiety. Thus, a fly’s willingness to overcome a bitter substance is a combination of its internal deprivation state, or drive, plus a peripheral reduction in the perception of the bitterness in the first place [14]. For the FLEA to be an improved measure of incentive motivation, we needed to show that the perception of the current would not change as a function of the internal feeding drive. Indeed, we found that increasing food deprivation from 6 to 18 h did not alter flies’ avoidance of higher current (Figs. 3d and 5b), while it did reduce their avoidance of bitter denatonium (Fig. 3a, b). Using the FLEA, we then found that both increasing an external incentive (higher sucrose concentration, Fig. 4) and increasing flies’ internal drive (longer food deprivation, Fig. 5c, d) would induce them to overcome a larger current to obtain a higher quality food source. This was true for two different wild-type strains, w* Berlin and *Canton-S*. In a single-choice setting, flies showed shorter duration proboscis events with lesser food deprivation (Fig. 1a), decreasing sucrose concentration (Fig. 1b), and increasing current (Additional file 3: Fig. S3). In a two-choice setting, changes in the 2nd well could also impact the proboscis event duration on a constant 1st well (Fig. 6c), suggesting that flies in the FLEA assay do evaluate and compare both offerings to make their feeding decisions. Future experiments will determine how calories vs. taste contribute to these feeding decisions. Taken together, the FLEA therefore represents an improved *Drosophila* assay that can be used to quantify incentive motivation in this highly manipulable model organism.

The NPF neuropeptide has previously been shown to be important for larvae to overcome bitter-laced food [15]. We replicated these results in adult flies, where we found that reduced NPF signaling made flies less willing to overcome bitter caffeine to obtain a preferable sucrose solution (Fig. 7c). However, when we performed the equivalent experiment with high-sucrose/high-current vs. low-sucrose/low-current pairings, loss of NPF had the opposite effect and made flies more willing to overcome higher current (Fig. 7a). Control experiments with 3 distinct NPF manipulations revealed that decreased NPF signaling reduced flies’ reaction to current (Fig. 7). Our findings do not invalidate previous findings indicating that NPF is involved in overcoming aversive stimuli in order to get superior food. However, we were unable to assess this, as we discovered here that NPF is required for normal reaction to electroshock. The FLEA therefore revealed a novel function for this neuropeptide. In addition to NPF’s involvement in feeding motivation and overcoming bitterness [15], NPF neurons are involved in sucrose sensing, where reduced activity decreased flies’ sensitivity to sugar [13]. This, again, would lead us to expect flies with reduced NPF function to become more, not less, sensitive to current, since it is the ratio of sucrose to current sensation that determines a feeding well’s attractiveness (Fig. 4). NPF also acts as a gate for the retrieval of appetitive memories, ensuring that flies remember these memories when they are hungry [16]. Furthermore, flies prefer to spend time in a place where their NPF neurons are optogenetically activated [21]. All these results are consistent with the model that NPF is largely involved in motivation for positively reinforcing behaviors. However, male flies that are sexually frustrated—by lack of mating and continued rejection from already mated females—prefer to drink more alcohol [22]. In that paradigm, frustrated males drink even more if they lack NPF signaling, and increased NPF signaling reduces their alcohol preference compared to controls. Thus, NPF might mediate alcohol aversion [6, 22], possibly by enhancing the sensation of aversive stimuli. An involvement for NPF in non-appetitive sensation/processing is also suggested by experiments showing that the L1 NPF neurons are involved in peripheral olfactory sensitivity to ethyl butyrate [23]. Thus, NPF is involved in sensory processing of a second modality, in addition to sugar taste [13]. Out of the ~ 32 NPF neurons in the adult brain, others (called DM) are involved in behavioral reinforcement [21]. Many of the NPF neurons project to the fan-shaped body (FSB) [21], and we recently showed that the FSB is involved in alcohol aversion [24]. Furthermore, FSB neurons are activated by acute electroshock [25]. It remains to be seen whether it is an FSB-subset of NPF neurons that are involved in the perception of electroshock. The mammalian ortholog of NPF, NPY, is well known to be involved in analgesia. For example, food deprivation induces NPY, which then suppresses inflammatory pain in mice’s paws [26]. However, acute mechanical pain suppresses these same NPY neurons [26]. The relationship between NPY and nociception is therefore complex, and it remains to be determined how *Drosophila* NPF is involved in the perception or processing of aversive electric shock.

## Conclusions

*Drosophila* has been a useful genetic organism to understand many neurological processes and behaviors [27]. Here, we describe a novel FLEA assay that allows us to ask what external and internal signals prod flies to overcome such electric shock. This assay will be useful to measure incentive motivation of this genetic model organism and to understand the genes and circuits that contribute to this disease-relevant behavior.

## Methods

### Fly husbandry and behavior

Male flies, age 2–8 adult days, were used for all experiments. Flies were grown and kept on standard cornmeal/agar medium at 25 °C with 70% relative humidity. Male w* Berlin flies were used as our wild-type strain. In some experiments, male *w*^*+*^ *Canton-S* flies were used as an additional wild-type strain. Transgenic flies were outcrossed to the w* Berlin genetic background for at least 5 generations. Food deprivation was done in vials containing 0.7% agar only, as a water source. Freely feeding single flies were filmed in a petri dish with a liquid sucrose drop containing 0.3% blue #1 to ensure we only analyzed the first feeding bout when scoring the movies. The FLIC assays were performed as described [10], with ten flies per arena. FLEA data was acquired from arenas with 2 wells per arena. Experimenters were blinded to distinct genotypes.

### FLEA system overview

The FLEA system is comprised of five components: feeding unit, resistor modules, data acquisition device, NI LabVIEW software, and analysis software in R. The first component, the feeding monitoring unit, is composed of a conductive metal baseplate, plastic reservoir, and printed circuit board. It houses eight feeding wells to conduct behavioral experiments. The second component, the resistor modules, is responsible for supplying current to each well in the feeding monitoring unit. The resistor modules are customizable to each of the 8 feeding wells and can be modularly exchanged. The third component, NI USB 6001 DAQ, is responsible for detecting analog signals from eight wells and forwarding the signal to the fourth component, NI LabVIEW software. The LabVIEW data acquisition software, NI SignalExpress, allows modification and customization of all the parameters of the system and records the data. For the last component, the R statistical analysis software is used for analyzing and visualizing the data and time preference analysis for behavioral experiments.

### FLEA hardware

The behavior board are composed of an aluminum plate, a plastic food reservoir, and a plastic cover based on the design of the FLIC, see Additional file 2: Fig. S2. A custom-made circuit board (AutoDesk EAGLE design .brd files available upon request) allows for the use of 8 wells and has receiving slots for 4 current resistor modules. The printed circuit board, the resistor modules, and the NI USB 6001 DAQ contain all the electronics needed for signal recognition, power supply to the board, and signal forwarding contacts to a computer for data acquisition. The printed circuit board contains two non-inverting operational amplifiers to provide a systemic gain of approximately 1.2, and it also contains 2 × 6 board-to-board male contacts for the attachment of resistor modules to the printed circuit board. The resistor module is the component that supplies current to each well. It contains two customizable current-limiting resistors, one for each well of a 2-well choice arena, and four gain resistors: two per well. The current-limiting resistors are used to regulate the amount of electrical current permitted to pass through the flies.

### FLEA signal processing and analysis

FLEA raw signal data was sampled at 500 Hz, or 100 Hz for experiments ≥ 1 h. A simple low-pass filter was utilized with the window size of 100 to reduce noise. Then, one mean data point was generated from the filtered signal to reduce the sampling rate from 500/100 Hz to 5 Hz. The process of filtering and sample rate reduction results in better resolution than sampling at 5 Hz. Then, filtered data was converted to the same scale as FLIC reading intensities ranging from 0 to 1023. The conversion factor for each current-limiting resistor was determined by measuring signal amplitude from circuits closed by defined resistors, standing in for flies. Baseline intensity varies linearly and/or non-linearly through time. For a linear baseline, the baseline estimation was performed by computationally estimating zero-slope baseline. For a non-linear baseline, we implemented a non-linear, non-parametric baseline adjustment algorithm—local polynomial regression (Loess [28])—to computationally estimate the baseline. Loess is a locally weighted polynomial regression performed via iterations of an M-estimation procedure with tricube kernel and Tukey’s biweight function as weighting parameters for time and intensity [28]. Window span and polynomial degree of regression were specified independently for each dataset. Baseline correction of FLEA data was then performed by subtraction of the estimated baseline. Residuals of baseline estimation were removed by zeroing values less than 4au intensity.

Each detected peak was classified as a leg event (LE, maximal intensity < 100) or proboscis event (PE, maximal intensity ≥ 100). Additionally, we identified exclusion criteria to eliminate false-positive events (like food splatter) and device errors and to better correlate feeding events with flies’ observed behavior. Exclusion criteria for the number of events per assay were derived from large pooled datasets from various conditions with the cutoffs based on the mean ± 2.5 standard deviations (e.g., between 4 and 212 events for a 30-min assay). Similarly, the exclusion criteria for event duration were calculated based on the third quartile plus 2× the interquartile range (which came to 4 and 40 s, for leg and proboscis events, respectively).

### Data analysis and statistics

The FLIC data was analyzed as described [10]; in brief, a signal of amplitude > 100 was designated a proboscis event, and smaller amplitude interactions were deemed leg events. We also filmed flies in the FLIC and a correlation of the filmed behavior with the obtained FLIC signal and designations suggested a sensitivity and specificity of ~ 90% for distinguishing leg from proboscis events. In the FLEA, the signal amplitude is changed as a function of the current-limiting resistor, and we normalized the data accordingly, such that we obtained the same 0–1023 data range. The *Interaction Time Preference Index* was calculated by taking the difference in total time interacted between the two wells and dividing it by the sum of the time interacted with both wells. This yields an index of 0, if both wells are equally interacted with, and + 1 or − 1 if only one well was interacted with exclusively. All data were checked for normality using Prism 8 (GraphPad Software Inc., San Diego, CA). Data were not normally distributed, and we excluded outliers for data sets with *n* > 8 if they fell 1.5× the interquartile range outside of the upper and lower quartiles. Data were compared using Mann-Whitney *U* tests, for pairwise 2-tailed comparisons, and Kruskal-Wallis tests with Dunn’s correction for multiple comparisons. Log-transformation of event duration rendered event duration data mostly normal, and we therefore used these transformed data to run 2-way ANOVAs for the data in Fig. 6.

## Supplementary Information

**Additional file 1: Fig S1.** Frequency plot of inter-event intervals from the FLIC (related to Fig. 1d). We chose 5 s, the inflection point of this distribution, to group events together, or apart.

**Additional file 2: Fig S2.** FLEA schematics: (Top left) FLEA setup, with board on top, exchangeable resistor module on bottom right, and general setup on the bottom left. The circuit diagrams for the resistor module (top, right) and FLEA board (bottom, front and back of board) are show.

**Additional file 3: Fig S3.** Proboscis events as a function of current/resistor: (Left) Average proboscis event duration decreases with current (*p* < 0.0001 for main effect), although there is no difference between 10 and 3 MΩ (*p* > 0.999; **p < 0.01 vs. 33 MΩ; *n* = 335–385 for 3–33 MΩ, 2 for 1 MΩ, from 16 to 28 wells; one-way ANOVA with Kruskal-Wallis post hoc test with Dunn’s multiple comparison). (Right) The frequency of proboscis events also decreases with increased current (***p < 0.0001 for one-way ANOVA main effect, *p* < 0.01 for each of 6 pairwise comparisons, *n* = 16–24 wells; Tukey post hoc comparisons).

**Additional file 4.** All the data from Figs. 1, 2, 3, 4, 5, 6 and 7, S1, S3.

## Data Availability

All the fly strains used, FLEA board design files, and setup information are available on request. The data analyzed during this study are included in this published article in Additional File 4: FLEA_Data_File.xlsx.
