# Supplementary figures and images for: The fly liquid-food electroshock assay (FLEA) suggests opposite roles for neuropeptide F in avoidance of bitterness and shock
Source: BMC Biol. 2021 Feb 16;19:31. doi: 10.1186/s12915-021-00969-7 (PMC7888162; doi:10.1186/s12915-021-00969-7)

inter event interval

*number of values*

200

100

0

1

3

5

7

9

11

13

15

17

19

21

Bin Center (sec)

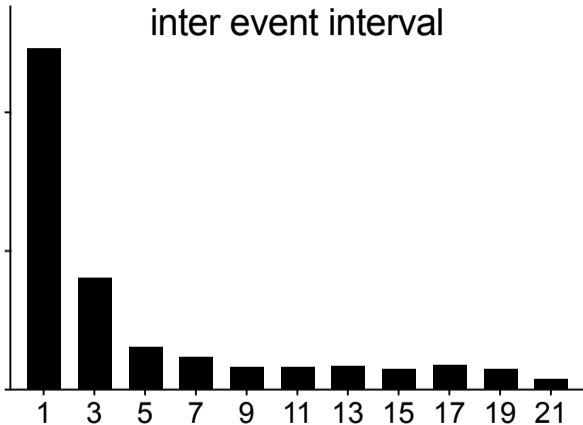

Supplement: Supplementary file 1 — Additional file 1: Fig S1. Frequency plot of inter-event intervals from the FLIC (related to Fig. 1d). We chose 5 s, the inflection point of this distribution, to group events together, or apart. [file 12915_2021_969_MOESM1_ESM.pdf]

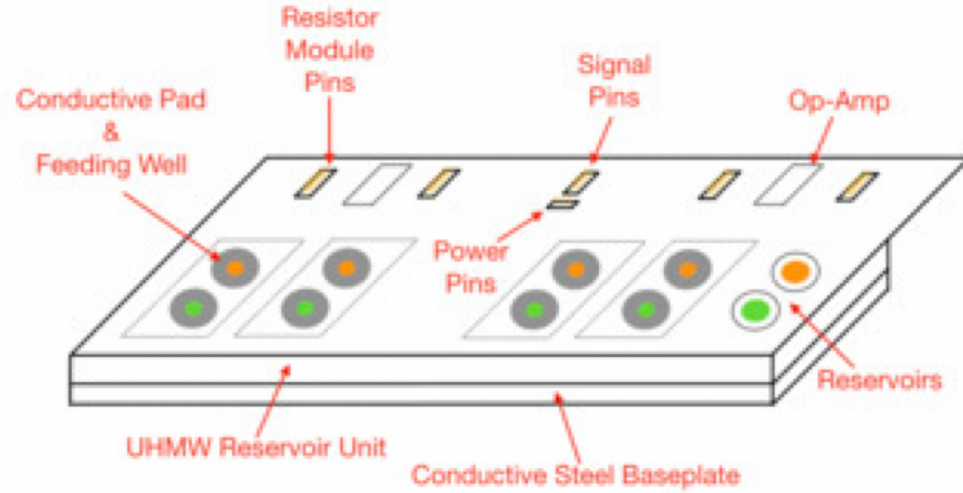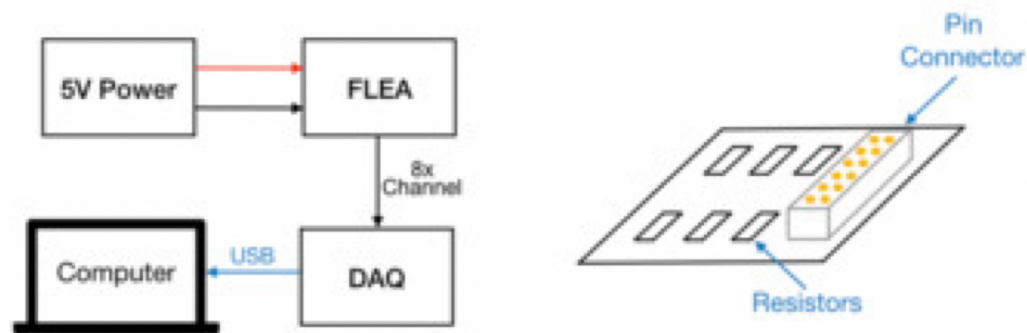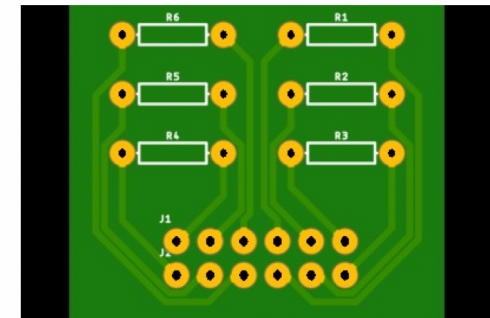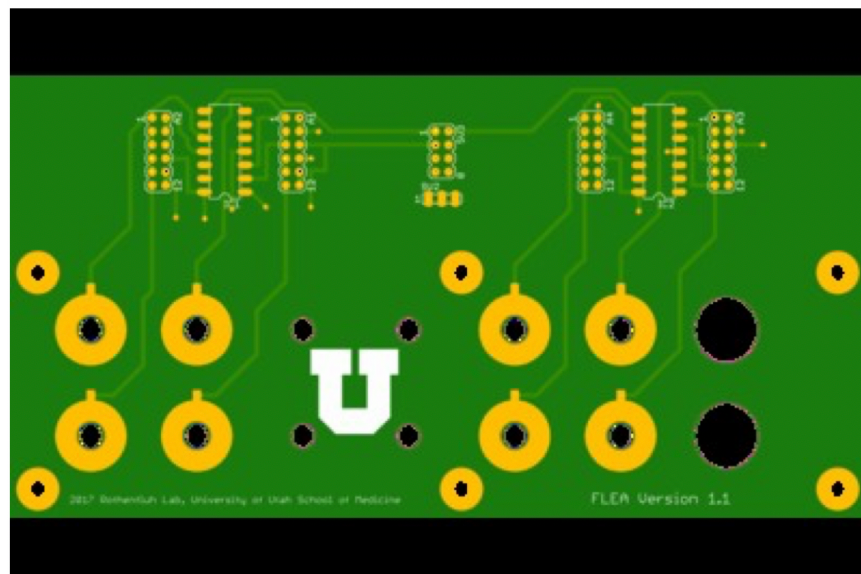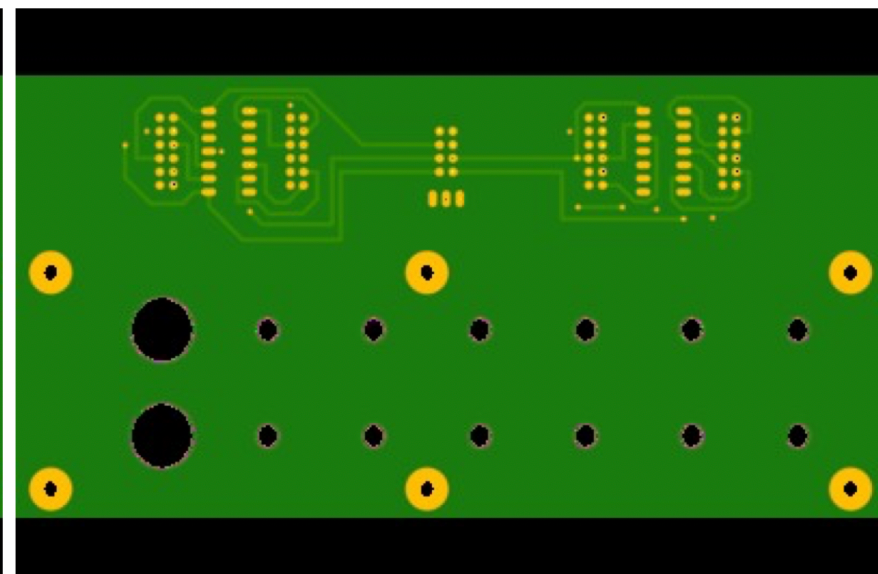

Supplement: Supplementary file 2 — Additional file 2: Fig S2. FLEA schematics: (Top left) FLEA setup, with board on top, exchangeable resistor module on bottom right, and general setup on the bottom left. The circuit diagrams for the resistor module (top, right) and FLEA board (bottom, front and back of board) are show. [file 12915_2021_969_MOESM2_ESM.pdf]

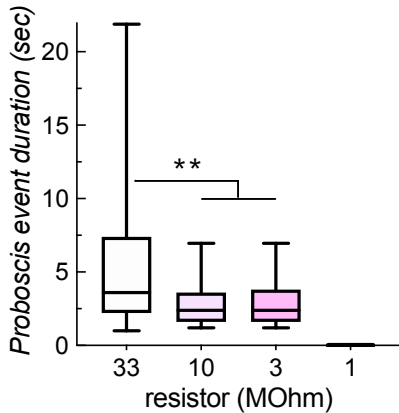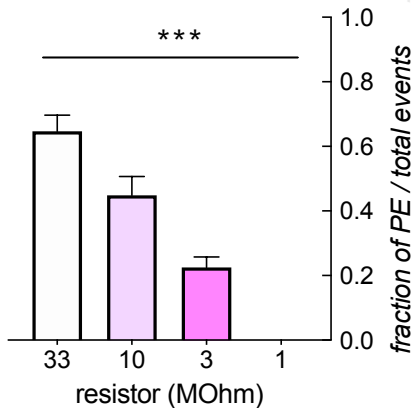

Supplement: Supplementary file 3 — Additional file 3: Fig S3. Proboscis events as a function of current/resistor: (Left) Average proboscis event duration decreases with current (p < 0.0001 for main effect), although there is no difference between 10 and 3 MΩ (p > 0.999; **p < 0.01 vs. 33 MΩ; n = 335–385 for 3–33 MΩ, 2 for 1 MΩ, from 16 to 28 wells; one-way ANOVA with Kruskal-Wallis post hoc test with Dunn’s multiple comparison). (Right) The frequency of proboscis events also decreases with increased current (***p < 0.0001 for one-way ANOVA main effect, p < 0.01 for each of 6 pairwise comparisons, n = 16–24 wells; Tukey post hoc comparisons). [file 12915_2021_969_MOESM3_ESM.pdf]
